# Decline in seminal quality in Indian men over the last 37 years

**DOI:** 10.1186/s12958-018-0425-z

**Published:** 2018-10-23

**Authors:** Priyanka Mishra, Mahendra Pal Singh Negi, Mukesh Srivastava, Kiran Singh, Singh Rajender

**Affiliations:** 1 Male Reproductive Biology Laboratory, Division of Endocrinology, Central Drug Research Institute, Lucknow, UP India; 2 Division of Toxicology and Experimental Medicine, Central Drug Research Institute, Lucknow, UP India; 3 Department of Molecular and Human Genetics, Banaras Hindu University, Varanasi, UP India

**Keywords:** Sperm concentration, Fertility, Seminal parameters, Indian men, Semen quality

## Abstract

**Background:**

Since the first report of a decline in semen quality in 1974, there have been several reports of similar declines across populations. Despite some scattered reports of declining semen quality in the Indian sub-continent, comprehensive studies analyzing semen quality over the last few decades have not been undertaken. We undertook the present study to investigate the temporal trend in semen parameters in Indian populations over a period of 37 years (1979–2016).

**Methods:**

Publications providing semen analysis details for fertile and infertile men from the Indian sub-continent were collected by a thorough literature search. Semen quality data for 6466 normal fertile or presumptive normal men (from 119 studies/data sets) and 7020 infertile men (from 63 studies/data sets) published between 1979 and 2016 were retrieved. We undertook systematic review and quantitative analysis of mean sperm count, motility, normal morphology and other available parameters. Data were analyzed to estimate semen parameters reference values for Indian men and to assess temporal trends in infertile, fertile and all subjects.

**Results:**

Seminal quality shows a decreasing temporal trend and the decrease is higher in infertile than fertile males. In pooled analysis for all individuals, significant (*p* < 0.05 or < 0.001) declines in sperm concentration and normal morphology are observed; however, isolated analysis for each group shows declines without statistical significance. The mean (± SD) semen volume, sperm concentration, total motility, rapid linear progressive motility, normal sperm morphology and sperm viability for Indian fertile men are 2.88 ± 0.77 ml, 81.08 ± 29.21 million/ml, 66.37 ± 10.95%, 52.64 ± 15.78%, 56.68 ± 20.23% and 72.63 ± 8.31%, respectively, whereas in infertile these are 3.07 ± 1.27 ml, 37.94 ± 26.41 million/ml, 40.22 ± 13.76%, 26.79 ± 15.47%, 36.41 ± 21.66% and 55.25 ± 11.99%, respectively. The mean seminal parameter values were significantly lower (*p* < 0.001) in infertile as compared to fertile men, except semen volume.

**Conclusions:**

Semen parameters in Indian men have declined with time and the deterioration is quantitatively higher in the infertile group. The study also provides reference values for semen parameters in Indian men.

## Background

The natural control for human fertility is largely vested in women. Women offer the rate-limiting step in fertility and fecundity because of the regulated production of eggs and other barriers during and around pregnancy. Men on the other hand are regular producers and always fertile during their reproductive life and can potentially father millions of children if fecundity is to be tested. A threat to sperm production would therefore be potentially dangerous for species renewal and propagation. Back in 1974, Nelson and Bunge while examining semen samples of 386 normal men consulting for vasectomy found that the average sperm count and average semen volume in these men were remarkably lower than the values reported by Macleod and Gold in 1951 [1, 2]. This was the first report of a decline in semen quality. The issue of declining semen quality got global attention in 1992 when a meta-analysis by Carlsen et al., revealed that the mean sperm concentration had fallen from 113 to 66 million**/**ml (almost 50%) in the past 50 years between 1938 and 1990 [3]. Subsequently, andrologists and clinicians all around the world conducted many retrospective and prospective studies to investigate such changes in the male population of their respective regions and many of them found conceivable downward trend in various semen parameters [4–18].

Concomitantly, it was found that fertility rates had declined [19, 20] and demand for artificial insemination had increased [21]. The effect of decline in semen quality can be understood more clearly from a decline in fertility rate seen in a number of populations. There is an average of less than two children per couple in Europe and Japan [22]. Similarly, in Spain and Italy, the average number of children per couple is less than 1.5 [23]. Similarly, in the United States, there have been appreciable declines in birth and fertility rates [24–26]. Most of the developed countries are either facing this problem or are approaching it at a faster pace [24]. With a decline in semen quality, there has been an increase in the demand for assisted reproductive techniques. International Committee for Monitoring Assisted Reproductive Technology in 2002 reported an increase in the use of intracytoplasmic sperm injection worldwide. According to the European IVF Monitoring Consortium, the number of reported cycles of IVF and ICSI has increased by 4.9% in comparison to 2011 [27]. In India, the number of IVF cycles increased at a rate of 18% and is expected to rise upto 20% by the year 2020 [28].

Despite some reports of declining semen quality in the Indian sub-continent [11, 29–31], comprehensive studies analyzing semen quality over the last few decades have not been undertaken. In the present study, we have attempted to find out the trend in semen quality among Indian men by analyzing semen quality data published over the last 37 years (1979 to 2016). The data over these years were compared in a retrospective longitudinal manner to find if the semen quality has quantitatively or qualitatively declined over a period of 37 years.

## Materials and methods

### Literature search and data collection

We undertook systematic review of literature and quantitative analysis of semen quality parameters. Relevant studies were gathered by computerized search on MEDLINE, GoogleScholar, ResearchGate and Scopus databases, using search terms such as sperm density, sperm morphology, sperm concentration, sperm motility, sperm count, male fertility and semen analysis. Inclusion criteria consisted of studies conducted on Indian male populations that had provided sufficient details about semen parameters of the cases and/or controls. Studies providing details only about cases or controls were also included. Exclusion criteria consisted of studies with predefined sperm count limits (cut off applied), as they would not be true representatives of semen parameters. No other limits were defined in selecting studies.

Standard format was used to tabulate the data thus collected. For each study, the year of publication, number of subjects, age, semen volume, sperm count (million per ml), total motility, rapid progressive motility, normal morphology, and sperm viability were recorded in a spreadsheet.

### Statistical analysis

Data were summarized as mean ± SD (standard deviation). Fertile and infertile groups were compared by Student’s independent ‘t’ test. Groups were also compared by two-way analysis of variance (ANOVA) and the significance of mean difference within and between the groups was tested by Newman-Keuls post hoc test after ascertaining normality by Shapiro-Wilk’s test and homogeneity of variances by Levene’s test. Pearson correlation analysis was undertaken to assess the association between variables. Simple linear regression analysis (regression coefficient: b) was undertaken to assess the rate of change (increase or decrease) in seminal parameters with time. A two-tailed (*α* = 2) *p* < 0.05 was considered to be statistically significant. Analyses were performed using STATISTICA software (Windows version 7.1, StatSoft, Inc., USA).

## Results

Data on various seminal parameters for 6466 normal or presumptive normal men (from 119 studies/data sets) and 7020 infertile men (from 63 studies/data sets) including our unpublished study cohorts were retrieved from articles published between 1979 and 2016. Thus, we included semen parameters for a total of 13,486 individuals. Most of the studies had reported sperm count and motility, while reporting of other data points varied significantly across studies. The available data points for each study were taken into account. For each variable, the mean value was calculated from the values reported across studies.

### Comparison of infertile versus fertile

The seminal qualities of fertile (*n* = 119 studies) and infertile (*n* = 63 studies) groups are summarized in Table 1. Comparing the mean (± SD) seminal quality of two groups, Student’s t test showed significantly different and lower sperm concentration (81.08 ± 29.21 vs. 37.94 ± 26.41, *t* = 9.48, *p* < 0.001), total motility (66.37 ± 10.95 vs. 40.22 ± 13.76, *t* = 11.88, *p* < 0.001), rapid linear progressive motility (52.64 ± 15.78 vs. 26.79 ± 15.47, *t* = 5.80, *p* < 0.001), normal morphology (56.68 ± 20.23 vs. 36.41 ± 21.66, *t* = 4.62, p < 0.001) and sperm viability (72.63 ± 8.31 vs. 55.25 ± 11.99, *t* = 5.59, p < 0.001) in infertile males as compared to fertile males (Table 1). However, no difference (*p* > 0.05) in semen volume was observed between the two groups. A large number of infertile individuals had sperm count, motility and morphology in the WHO normal range. The etiology of infertility in these individuals remained unknown and was not related to inadequate sperm production or lack of motility.

**Table 1:** Seminal quality of fertile and infertile Indian men

Variables	Fertile		Infertile		Total		*p* value (Fertile vs. Infertile)
	n	Mean ± SD, Range Median	n	Mean ± SD, Range Median	n	Mean ± SD, Range Median	
Semen volume (ml)	53	2.88 ± 0.77 (1.16–4.70) 2.90	22	3.07 ± 1.27 (1.50–7.60) 2.81	75	2.94 ± 0.94 (1.16–7.60) 2.85	0.431
Sperm concentration (million/ml)	112	81.08 ± 29.21 (22.00–175.00) 76.23	59	37.94 ± 26.41 (1.60–113.50) 32.16	171	66.20 ± 34.90 (1.60–175.00) 68.00	< 0.001
Total motility (%)	89	66.37 ± 10.95 (36.70–90.00) 66.80	44	40.22 ± 13.76 (7.50–82.66) 39.85	133	57.71 ± 17.15 (7.50–90.00) 58.60	< 0.001
Rapid linear progressive motility (%)	30	52.64 ± 15.78 (9.27–74.10) 57.02	21	26.79 ± 15.47 (1.40–51.14) 32.00	51	42.00 ± 20.13 (1.40–74.10) 41.20	< 0.001
Normal morphology (%)	62	56.68 ± 20.23 (17.00–98.00) 53.13	35	36.41 ± 21.66 (4.69–97.50) 30.81	97	49.37 ± 22.85 (4.69–98.00) 49.90	< 0.001
Viability (%)	30	72.63 ± 8.31 (54.90–90.81) 73.25	14	55.25 ± 11.99 (31.00–81.33) 55.33	44	67.10 ± 12.54 (31.00–90.81) 67.98	< 0.001

Data were expressed as n (number of studies/data sets), Mean ± SD (standard deviation), range (min-max) and median. Fertile and infertile groups were compared by independent Student’s t test

### Reference semen parameter values for Indian men

Despite significant number of studies available on semen analysis in fertile and infertile individuals, semen reference values for Indian men are not available. The WHO 2010 reference manual is based on semen parameters collected from various populations, but the data for Indian men were not considered for this evaluation. Nevertheless, it is well known that semen parameters vary significantly across various populations and the reference semen parameters for Indian population are not available. From this collection of data, we estimated values that could provide us with the reference semen parameters for normal (fertile) Indian men. The mean (± SD) semen volume, sperm concentration, total motility, rapid linear progressive motility, normal morphology and sperm viability for Indian fertile men are 2.88 ± 0.77 (ml), 81.08 ± 29.21 million/ml, 66.37 ± 10.95%, 52.64 ± 15.78%, 56.68 ± 20.23% and 72.63 ± 8.31%, respectively (Table 1). The 5th percentile values for Indian fertile men are semen volume 1.61 ml, sperm concentration 39.45 million/ml, total motility 49.15%, rapid linear progressive motility 20.20%, normal morphology 22.11% and viability 57.60% (Table 2).

**Table 2:** Different centile values for semen parameters in fertile men

Variables	N*	2.5	5	10	25	50	75	90	95	97.5
Semen volume (ml)	2824	1.27	1.61	2.00	2.38	2.90	3.26	3.90	4.46	4.65
Sperm concentration (million/ml)	6103	33.61	39.45	48.69	60.48	76.23	90.03	129.29	137.18	159.56
Total motility (%)	5343	46.75	49.15	53.84	57.00	66.80	74.03	80.60	85.51	88.90
Rapid linear progressive motility (%)	1584	9.27	20.20	29.36	41.66	57.02	66.24	69.07	73.33	–
Normal morphology (%)	3730	19.79	22.11	32.07	39.81	53.13	71.81	87.37	91.86	95.13
Viability (%)	2636	54.90	57.60	62.34	65.50	73.25	79.12	83.68	88.82	–

*Number of subjects

### Temporal trend in semen parameters

**Fig. 1:**
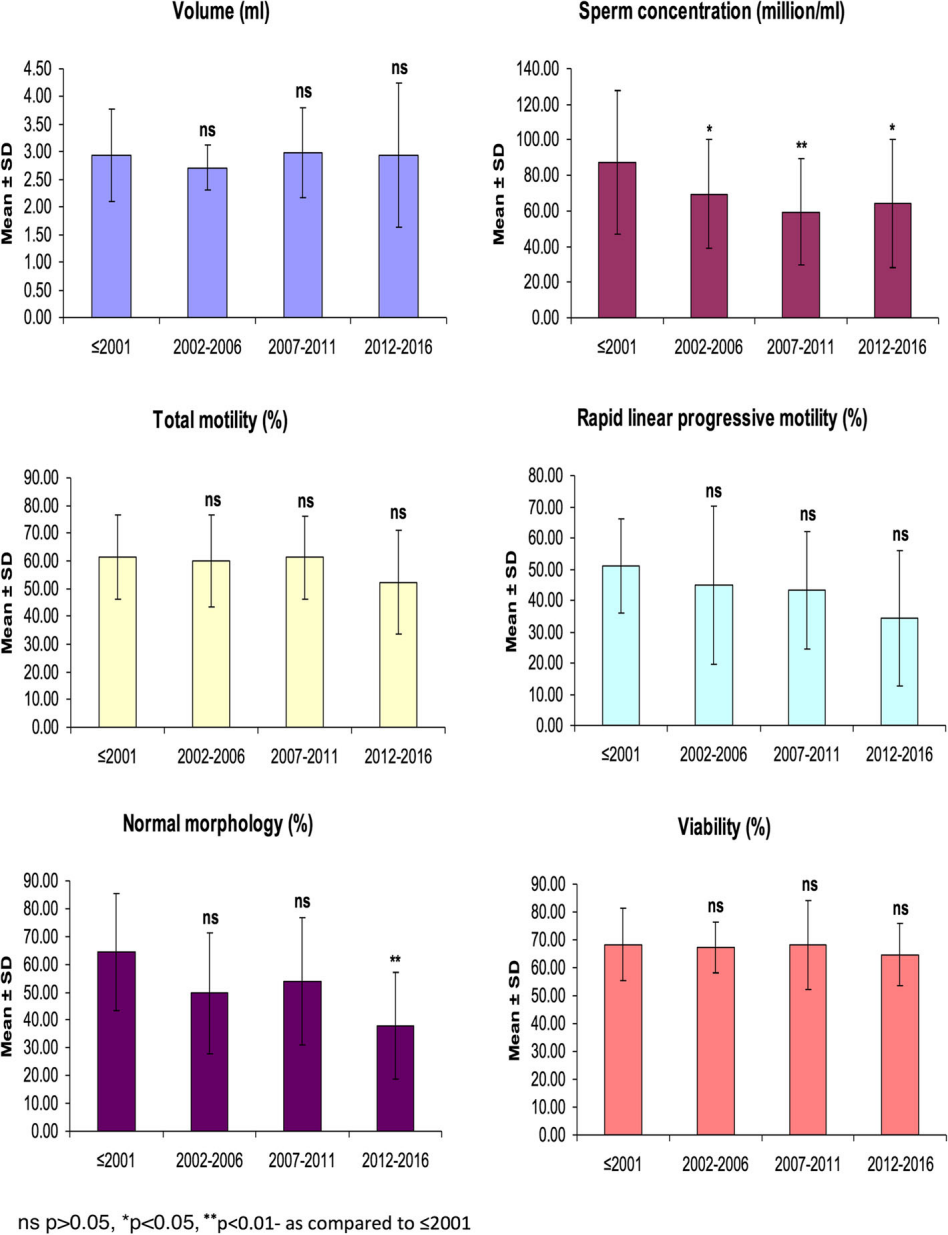
Temporal trend in mean semen parameters in the whole population (fertile plus infertile)

In total (fertile + infertile) population, the mean sperm concentration showed a significant (*p* < 0.05 or *p* < 0.001) decrease across all periods as compared to the baseline (before 2001) (Fig. 1 and Table 3). Further, the mean normal morphology also showed a significant (*p* < 0.05 or < 0.01) decrease in 2012–2016 period as compared to both before 2001 and 2007–2011 periods. Moreover, mean rapid linear progressive motility showed a decreasing trend with time, but the difference between these periods did not touch statistical significance (*p* > 0.05).

**Table 3:** Seminal quality in Indian fertile and infertile men over five-year periods

Variables	Fertile		Infertile		Total		p value (Fertile vs. Infertile)
	n	Mean ± SD	n	Mean ± SD	n	Mean ± SD	
Semen volume (ml):							
≤ 2001	8	2.74 ± 0.52	2	3.72 ± 1.73	10	2.93 ± 0.84	0.701
2002–2006	6	2.78 ± 0.42	2	2.50 ± 0.42	8	2.71 ± 0.41	0.896
2007–2011	23	2.97 ± 0.94	13	3.00 ± 0.56	36	2.98 ± 0.81	0.963
2012–2016	16	2.86 ± 0.74	5	3.21 ± 2.51	21	2.94 ± 1.30	0.941
Sperm concentration (million/ml):							
≤ 2001	21	93.39 ± 37.91	4	55.38 ± 43.51	25	87.31 ± 40.45	0.008
2002–2006	14	81.26 ± 24.73	6	42.43 ± 26.98	20	69.61 ± 30.72^*^	0.004
2007–2011	42	73.46 ± 22.80	27	37.05 ± 26.33	69	59.22 ± 29.98^**^	0.008
2012–2016	35	82.77 ± 30.20	22	34.65 ± 23.43	57	64.19 ± 36.31^*^	0.001
Total motility (%):							
≤ 2001	15	64.20 ± 13.84	2	41.05 ± 6.58	17	61.48 ± 15.15	< 0.001
2002–2006	12	67.12 ± 11.63	4	39.01 ± 9.39	16	60.09 ± 16.58	< 0.001
2007–2011	31	68.93 ± 9.48	18	47.93 ± 13.87	49	61.22 ± 15.13	0.003
2012–2016	31	64.56 ± 10.50	20	33.43 ± 11.62	51	52.35 ± 18.79	< 0.001
Rapid linear progressive motility (%):							
≤ 2001	4	54.02 ± 17.84	2	45.07 ± 8.58	6	51.04 ± 15.07	0.399
2002–2006	4	58.31 ± 13.42	2	18.00 ± 21.21	6	44.88 ± 25.13	0.009
2007–2011	13	57.17 ± 11.28	12	28.46 ± 12.79	25	43.39 ± 18.79	0.065
2012–2016	9	42.96 ± 19.21	5	19.01 ± 18.06	14	34.41 ± 21.67	0.069
Normal morphology (%):							
≤ 2001	13	70.29 ± 19.46	4	45.84 ± 14.66	17	64.54 ± 20.94	0.088
2002–2006	11	56.36 ± 19.08	4	31.28 ± 19.16	15	49.67 ± 21.69	0.054
2007–2011	16	59.86 ± 19.80	14	47.09 ± 24.55	30	53.90 ± 22.69	0.343
2012–2016	22	46.49 ± 16.98	13	23.59 ± 13.46	35	37.99 ± 19.18^**^	0.064
Viability (%):							
≤ 2001	6	73.71 ± 10.67	3	57.48 ± 10.93	9	68.30 ± 12.92	0.100
2002–2006	8	69.86 ± 7.96	2	56.90 ± 7.64	10	67.27 ± 9.25	0.154
2007–2011	9	75.63 ± 8.68	5	54.86 ± 18.17	14	68.21 ± 15.96	0.060
2012–2016	7	71.02 ± 6.05	4	53.24 ± 8.46	11	64.56 ± 11.13	0.122

Data were expressed as n (number of studies/data sets), Mean ± SD (standard deviation). Groups were compared by ANOVA followed by Newman-Keuls test. ^*^p < 0.05 0r ^**^p < 0.01- as compared to ≤2001 (total population)

The seminal qualities of fertile and infertile males were also individually compared on a five-year time interval basis (Table 3). The mean seminal quality (sperm concentration, total motility, rapid linear progressive motility, normal morphology and viability) showed a decreasing trend with time with a higher decrease in infertile than fertile males. Newman-Keuls test showed insignificant (p > 0.05) difference in the mean seminal parameters across periods in both groups. Sperm concentration and total motility differed significantly between the two groups and remained significantly lower (*p* < 0.01 or < 0.001) in infertile males as compared to fertile males across all periods.

### Rate of loss in seminal quality

**Fig. 2:**
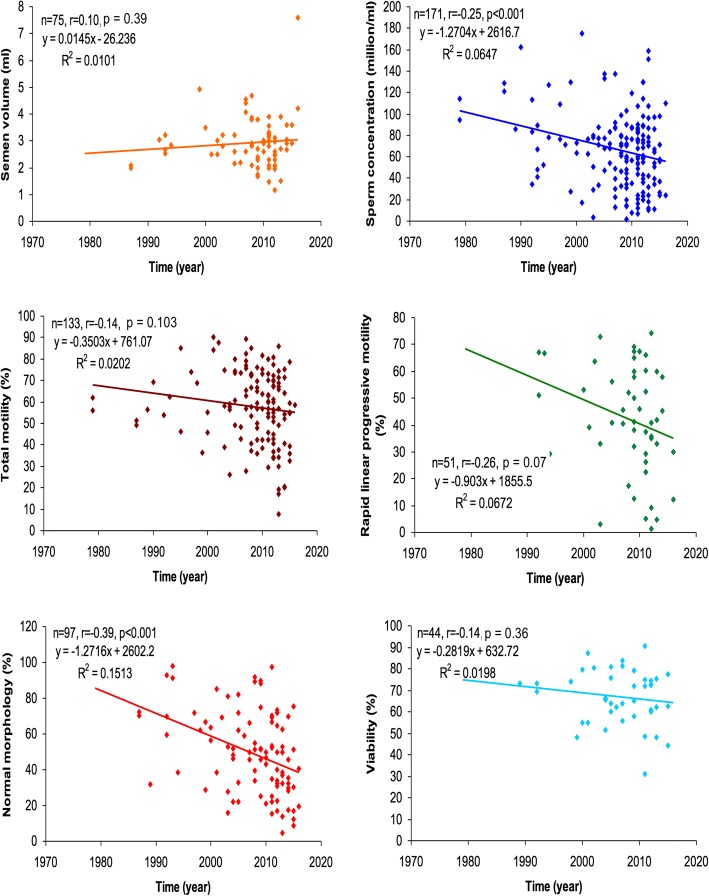
Correlation and regression analysis of temporal trend in semen parameters in total population (fertile plus infertile)

Correlation and regression analyses of seminal parameters for fertile, infertile and total population are summarized in Table 4. Except for semen volume, almost all parameters (the seminal quality) showed a negative (inverse) correlation with time in both the groups. In total population, sperm concentration (*r* = − 0.25, *p* < 0.001) and normal morphology (*r* = − 0.39, p < 0.001) showed a highly significant downward trend. Normal morphology (r = − 0.39, *p* < 0.01) in the fertile group, and sperm concentration (r = − 0.25, *p* < 0.05) and total motility (*r* = − 0.30, p < 0.05) in the infertile group, reached statistical significance (Fig. 2 and Table 4).

**Table 4:** Correlation and regression analysis between time and seminal parameter levels in Indian fertile and infertile men

Variables	Fertile				Infertile				Total			
	n	r	b	p value (t_b_)	n	r	b	p value (t_b_)	n	r	b	p value (t_b_)
Semen volume	53	0.12	0.012	0.408	22	0.06	0.020	0.778	75	0.10	0.015	0.391
Sperm concentration	112	−0.17	−0.635	0.071	59	−0.25^*^	−1.467	0.060	171	−0.25^***^	−1.270	< 0.001
Total motility	89	0.10	0.133	0.371	44	−0.30^*^	−1.112	0.046	133	−0.14	−0.350	0.103
RLPM	30	−0.23	−0.570	0.232	21	−0.40	−1.191	0.073	51	−0.26	−0.903	0.066
Normal morphology	62	−0.39^**^	−1.030	0.002	35	−0.29	−1.181	0.090	97	−0.39^***^	−1.272	< 0.001
Viability	30	0.00	0.003	0.989	14	−0.35	−0.672	0.213	44	−0.14	−0.282	0.362

*- p < 0.05, **- p < 0.01, ***- *p* < 0.001. **n =** number of observations, **r =** correlation value, **b =** regression coefficient value, RLPM: rapid linear progressive motility

Regression analysis showed a significant rate of decrease of 1.27 million/ml per year in sperm concentration (b = − 1.270, t_b_ = 3.42, p < 0.001), and 1.27% per year in normal morphology (b = − 1.272, t_b_ = 4.12, *p* = 0.002) in total population (Fig. 2 and Table 4). In the fertile group, normal morphology decreased at a rate of 1.030% per year (b = − 1.030, t_b_ = 3.28, p = 0.002), and in infertile group, total motility decreased at a rate of 1.11% per year (b = − 1.112, t_b_ = 2.06, *p* = 0.046) (Table 4). Moreover, the rate of decrease in semen volume, sperm concentration, total motility, rapid linear progressive motility, normal morphology and viability was 1.7, 2.3, 8.4, 2.1, 1.1 and 224 folds in the infertile group in comparison to the fertile group.

## Discussion

Over decades and centuries, humans have evolved with respect to food habits, life style and environment, resulting in significant changes in the incidence and prevalence of a number of diseases. Semen quality is generally not paid heed until it deteriorates to the level of infertility. Interestingly, the fertility rate has gone down in the last few decades and a number of populations have reached a stage where growth revival is difficult to achieve. Apart from a number of other contributing factors, deterioration in semen quality is the prime factor contributing to a drop in the fertility rate. Since the first report of a decline in semen quality by Nelson and Bunge in 1974, similar declines have been reported in various other populations [2]. Consistent downward trend was reported in American men seeking infertility advice [32]. Another study from Boston area reported declines in sperm count, total count and total motile count [17]. Nonetheless, no change in semen quality was reported in the USA by earlier studies [33–35]. Swan et al. (1997) extracted data for 56 studies from the United States, Europe and non-western countries and found significant declines in sperm density in the United States (1.5% per year), Europe and Australia (3% per year) [36].

Significant declines in sperm count (median values 73.4 million per ml in 1952 and 54.5 million per ml in 1972), motility and an increase in the number of abnormal spermatozoa have also been reported in Danish men [37]. Osser et al. (1984) reported that median sperm concentration in Sweden declined from 109 million per ml in 1960 to 65 million per ml in 1980 [38]. In European men, a time-dependent decline in sperm concentration over the last 50 years and an overall 32.5% decrease in mean sperm concentration have been reported recently [39]. Similarly, mean concentration of spermatozoa in Italy shrunk from 88 million per ml in 1981 to 61 million per ml in 1995, mean motility declined from 74 to 66% and typical morphology fell from 76 to 63% [40]. Slama et al. (2004) estimated a 21% (1977–1992) and 47% decrease (1947–1992) in sperm count in French sperm donors [41]. Sperm concentration dropped down from 27.75 million per ml in 1986 to 4.60 million per ml in 2003 in Austria [42]. In Finland, semen quality analysis during 1998–2006 found that young Finns showed lower sperm counts in the most recent birth cohort compared with few years older cohort [43].

Deterioration in semen parameters was also observed in Asia, in the cities of Japan, China, Israel, Korea and India. In the Indian region, Marimuthu et al. (2003) reported no change in semen quality among 1176 subjects attending infertility clinic at Delhi over a period of 11 years [30] whereas the study by Gopalkrishnan (1998) on fertile subjects from Mumbai suggested a significant decline in semen quality [29]. Similarly, another study by Mukhopadhyay et al. (2009) on 3729 males attending an infertility clinic in Calcutta between 1981 and 1985 and 2000–2006 found that semen volume and motility had declined significantly in that two decade period [31]. In the southern part of India, declines in motility and semen volume were observed between 1993 and 2005 [11]. Our analysis covers populations from all regions of India and suggests a significant decline in semen quality over more than three decades, with a more prominent deterioration in the infertile population.

WHO reference parameters are used to demarcate between fertile and infertile individuals. WHO, 2010 parameters collected data from various populations without contribution from the Indian subcontinent [44]. India being the second most populated country in the world can contribute significantly to the estimate of reference semen parameters. The present study is not an original data collection, but data from secondary sources present how semen parameters in Indian men look like. Since the studies included in this analysis were conducted on different populations across the Indian sub-continent, it should provide the closest reference values for Indian men. As far as the loss of semen quality in Indians is concerned, temporal analysis of pooled data for the overall population (fertile and infertile individuals) showed statistically significant negative trend in sperm concentration and normal sperm morphology. We found a downward trend in several important semen parameters like concentration, rapid progressive motility, and normal morphology of sperm in the whole population and the declines were more pronounced in infertile males. We found that a large number of infertile individuals have semen parameters in the WHO normal range. The etiology of infertility in these remains unknown and appears to be related to functional loss of sperm fertility. These are the most interesting cases for further research to uncover functional causes of male infertility.

While the declines in semen parameters are appreciable, the causes behind the same remain unknown. Modern life-style with high stress levels, smoking, drinking, lack of exercise, exposure to radiations and endocrine disrupting chemicals (EDCs) has contributed to declines in the seminal parameters. A rapid expansion of industries has added a number of hazardous chemicals into the environment, which affect semen parameters directly or indirectly. Occupational and domestic exposure to EDCs, of which the use of estrogenic chemicals is the most common, is a serious factor affecting semen parameters. Apart from the direct exposure, prenatal and postnatal exposures have also been suggested to affect sperm parameters. A follow-up study over two decades suggested that alcohol intake during pregnancy correlates with lower sperm count in sons [45]. Similarly, maternal smoking during pregnancy has also been reported to result in poor sperm parameters in sons [46]. Not only maternal, but also paternal smoking has been shown to affect semen quality in the coming generations [47]. Apart from the above, inhalation or absorption of phthalate compounds during pregnancy causes birth of male children with poor sperm parameters and testosterone production [48]. Recent studies have suggested that endocrine disrupting chemicals, smoking, alcohol, and occupational or domestic exposures all can have trans-generational effects and adversely affect semen parameters for several generations. In the developing countries like India, very high level of pollution [49] and the ubiquitous presence of endocrine disrupting chemicals in the environment [50] contribute significantly to the decline in fertility parameters [51].

## Conclusions

In conclusion, seminal quality in Indian men showed a decreasing temporal trend with a significantly much higher decrease in infertile males than their fertile counterparts. In pooled analysis for all individuals, significant declines in sperm concentration and normal morphology were observed; however, isolated analysis for each group showed decline without statistical significance. Average semen parameter values for normal (fertile) Indian men are: volume 2.88 + 0.77 ml, sperm concentration 81.08+ 29.21 per ml, sperm motility (total) 66.37+ 10.95%, rapid linear progressive motility 52.64 + 15.78%, normal morphology 56.68 + 20.23%, and viability 72.63 + 8.31%. The 5th percentile values for semen volume, sperm concentration, total motility, rapid linear progressive motility, normal morphology and sperm viability are 1.61 ml, 39.45 million/ml, 49.15%, 20.20%, 22.11% and 57.60%, respectively. These can be taken as reference values for semen parameters in Indian men.
